# A Complex Case of Retinoblastoma Solved by the Combined Approach of Humor/Plasma cfDNA-NGS and LR-WGS

**DOI:** 10.3390/genes16121399

**Published:** 2025-11-22

**Authors:** Simona Innamorato, Simona L. Basso, Omaima Belakhdar, Mirella Bruttini, Chiara Fallerini, Heyran Huseynli, Giulia Caccialupi, Elena Pasquinelli, Mariarosaria Adduci, Giorgio Signori, Felice Arcuri, Valeria Malagnino, Maria Chiara Siciliano, Stefano Lazzi, Simone Pesaresi, Daniela Galimberti, Paolo Galluzzi, Sonia De Francesco, Theodora Hadijstillanou, Anna Maria Pinto, Alessandra Renieri, Francesca Ariani

**Affiliations:** 1 Medical Genetics, University of Siena, 53100 Siena, Italy; simona.innamorato@dbm.unisi.it (S.I.); francesca.ariani@unisi.it (F.A.); 2 Department of Medical Biotechnologies, Med Biotech Hub and Competence Center, University of Siena, 53100 Siena, Italy; 3 Genetica Medica, Azienda Ospedaliero-Universitaria Senese, 53100 Siena, Italy; 4 Unit of Pathological Anatomy, Azienda Ospedaliera Universitaria Senese, 53100 Siena, Italy; 5 Unit of Pediatrics, Department of Maternal, Newborn and Child Health, Azienda Ospedaliera Universitaria Senese, 53100 Siena, Italy; 6 Unit of NeuroImaging and NeuroIntervention, Azienda Ospedaliera Universitaria Senese, 53100 Siena, Italy; 7 Unit of Ophthalmology, Department of Medicine, Surgery and Neuroscience, Azienda Ospedaliera Universitaria Senese, 53100 Siena, Italy

**Keywords:** rare genetic diseases, cfDNA, liquid biopsy, next-generation sequencing, bioinformatics, long-read–whole-genome sequencing, single-nucleotide variants, variant interpretation, structural variants, genetic diagnosis

## Abstract

Background: Complex cases of retinoblastoma (RB) often require integrative molecular approaches to define tumor etiology and guide clinical management. Purpose: Our aim was to evaluate the usefulness of combining aqueous humor (AH)/plasma cell-free DNA next-generation sequencing (cfDNA-NGS) and long-read–whole-genome sequencing (LR-WGS) to resolve diagnostically challenging RB cases. Case Description: We report the case of a 3-year-old Caucasian girl, conceived by heterologous assisted reproductive technology (ART), presenting with unilateral, widely infiltrative RB in the right eye. She exhibited limited verbal communication, a glabellar angioma extending to the nasal bridge and philtrum, and mild hypertelorism. Standard blood testing revealed no pathogenic SNVs, CNVs, or methylation abnormalities in the *RB1* gene. Targeted cfDNA analysis using the Illumina TruSight Oncology 500 (TSO500) panel on AH and plasma identified a somatic *RB1* splice-site variant (c.1498+2T>C) with a variant allele frequency (VAF) of 98.5%, consistent with biallelic inactivation. Additional gains (fold change > 1.5) were found in AH and confirmed in plasma, suggesting a germline 13q duplication. Third-generation LR-WGS, performed with Oxford Nanopore Technology (ONT), on blood confirmed a 24.6 Mb duplication on chromosome 13, compatible with the rare 13q duplication syndrome characterized by psychomotor delay, craniofacial dysmorphism, and hemangiomas. AH-cfDNA revealed additional somatic copy-number alterations, including amplifications (i.e., *MDM4* and *ALK*) and deletions (i.e., *BRCA2*), indicating progressive clonal tumor evolution. Conclusions: This experience tells us that a combined approach with TSO500 Illumina NGS on cfDNA, along with LR-WGS, is able to help solve complex cases and define the appropriate treatment and surveillance strategy.

## 1. Introduction

Retinoblastoma (RB) is a rare pediatric tumor of the retina occurring in approximately 1 per 16,000–24,000 live births [1]. Despite advances in treatment, such as intra-arterial chemotherapy, which have significantly improved patient outcomes, tumor recurrence remains quite common, highlighting the need for new biomarkers to monitor disease progression [2]. Loss of function of the *RB1* tumor suppressor gene is a pivotal factor in the pathogenesis of RB. This loss may occur through several mechanisms, including point mutations, deletions, or chromosomal rearrangements. Biallelic inactivation of *RB1* results in a benign *RB1*^−^/^−^ retinoma, and full malignancy is acquired after additional recurrent events, including gain of 1q, 2p, 6p, and 13q and loss of 16q [3]. The *RB1* gene encodes the retinoblastoma protein (pRB), a nuclear phosphoprotein that acts as a key regulator of the G1/S checkpoint by binding and inhibiting E2F transcription factors. When hypophosphorylated, pRB represses transcription of genes required for DNA synthesis, maintaining the cell in G1; phosphorylation by cyclin-dependent kinases (CDK4/6 and CDK2) releases this inhibition, allowing cell-cycle progression [4]. Beyond this canonical role in cell-cycle control, recent discoveries have revealed that pRB functions as a multifaceted tumor suppressor involved in chromatin organization, DNA repair, differentiation, and metabolic homeostasis [4,5]. For instance, pRB interacts with histone modifiers and chromatin remodelers to preserve genomic stability and proper transcriptional silencing [5,6]. Moreover, pRB plays a critical role in maintaining the differentiated state of retinal progenitor cells, and its loss promotes lineage plasticity—the ability of tumor cells to change identity and adopt alternative differentiation programs that favor tumor progression [6]. From a therapeutic perspective, these recent mechanistic insights into pRB function have also highlighted potential pharmacological vulnerabilities. Synthetic-lethal interactions have been described between *RB1* loss and mitotic kinases such as Aurora kinase A (AURKA), whose inhibition selectively induces mitotic catastrophe in *RB1*^−^/^−^ cells [7]. Notably, this approach has shown promise in RB models with concomitant *MYCN* amplification, where AURKA inhibition produced strong anti-tumor effects [8]. Similarly, the use of CDK4/6 inhibitors and DNA-repair pathway modulators is being explored to restore cell-cycle control or exploit vulnerabilities associated with pRB dysfunction [9]. Building on these molecular insights, recent genomic and transcriptomic studies have revealed that RB is not a genetically uniform disease but rather includes molecularly defined forms characterized by specific oncogenic drivers and chromosomal alterations. Understanding these differences has become increasingly relevant for both prognostic assessment and the development of tailored diagnostic and therapeutic approaches. Distinct molecular subtypes of RB exist, offering potential avenues for individualized therapeutic interventions [10]. A rare form of RB was first reported in 2013, in which high-level amplification of the oncogene *MYCN* drives tumorigenesis in the absence of pathogenic *RB1* mutations (*MYCN*-amplified, *RB1*^+^/^+^ retinoblastoma). This molecular subtype, accounting for roughly 1–2% of cases, represents an alternative oncogenic route that bypasses pRB loss and directly promotes uncontrolled proliferation of retinal progenitor cells [11]. Later investigations revealed that chromothriptic rearrangements of chromosome 13 can disrupt the *RB1* locus, resulting in its functional loss through a single catastrophic genomic event [12].

Detection of circulating tumor DNA (ctDNA) has led to a promising, active area of translational research in oncology. A liquid biopsy approach overcomes many difficulties and risks associated with obtaining traditional tissue biopsies, and it is especially critical in cancers like RB, wherein direct tissue biopsy is strictly contraindicated for risk of tumor dissemination. In this setting, analysis of cell-free DNA (cfDNA) from the aqueous humor (AH) has emerged as a more informative and disease-relevant source than plasma for liquid biopsy analyses [13]. Independent groups in Los Angeles and Birmingham subsequently demonstrated that cfDNA extracted from the AH faithfully reflects the tumor genome, enabling detection of somatic copy number alterations (SCNAs) and other pathogenic variants in RB [14,15]. Until 2017, studies of AH focused exclusively on enucleated eyes, making clinical correlations impossible. The landmark study by Berry et al. introduced AH sampling during eye-salvage therapy, establishing its prognostic and monitoring value through cfDNA analysis [13]. Gain of chromosome 6p emerged as the most recurrent SCNA identified in enucleated eyes and has been consistently linked to unfavorable ocular prognosis [16].

In this context, we describe a complex case of RB in which a multimodal genomic approach—including AH and plasma cfDNA liquid biopsy, together with LR-WGS—provided new insights into the molecular mechanisms underlying RB development.

## 2. Materials and Methods

### 2.1. Patient Enrolment

This study includes a patient who was diagnosed with RB in December 2023. The family underwent genetic counseling and provided written informed consent for the diagnostic use of DNA samples at the Medical Genetics Unit, Azienda Ospedaliera Universitaria Senese (Siena, Italy).

### 2.2. Genomic DNA Isolation and Analysis

Genomic DNA was extracted from peripheral blood collected in EDTA tubes using the MagCore^®^ Super Automated Nucleic Acid Extractor (Diatech Lab Line, Jesi, Italy). DNA concentration and purity were assessed spectrophotometrically using a NanoDrop 2000 instrument (Thermo Fisher Scientific, Waltham, MA, USA). The A260/A280 ratio was used to verify DNA quality before downstream applications.

### 2.3. Clinical Exome Sequencing (CES)

Clinical Exome Sequencing (CES) was performed using the TruSight One Expanded Sequencing Panel (Illumina, San Diego, CA, USA), which targets 6794 clinically relevant genes. Library preparation was carried out with the Nextera Flex for Enrichment kit following the manufacturer’s protocol. Sequencing was performed on the NovaSeq 6000 platform (Illumina). Sequencing reads were aligned to the UCSC/hg19 human reference genome, and variant calling and annotation were conducted using the eVai platform (enGenome Srl, Pavia, Italy) for variant prioritization and clinical interpretation.

### 2.4. Methylation-Specific Multiplex Ligation-Dependent Probe Amplification (MS-MLPA) Analysis

MS-MLPA was performed using the ME028-RB1 kit (MRC Holland, Amsterdam, The Netherlands), according to the manufacturer’s instructions. The kit includes methylation-sensitive probes targeting the *RB1* promoter region (CpG106) and the imprinted locus (CpG85). Data analysis and copy number interpretation were carried out with Coffalyser.Net software version v.250317.1029 (MRC Holland, Amsterdam, The Netherlands).

### 2.5. Cell-Free fDNA (cf-DNA) Isolation and Next-Generation Sequencing (NGS) Liquid Biopsy Analysis

**Figure 1:**
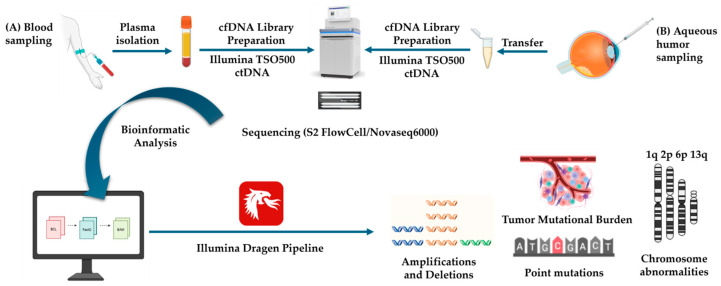
NGS-cfDNA liquid biopsy workflow in the retinoblastoma case: (**A**) Blood sampling; (**B**) aqueous humor sampling at enucleation. TSO500 ctDNA Illumina analysis containing 523 cancer driver genes and sequencing on a Novaseq6000 apparatus. Illumina Dragen Bioinformatics Pipeline enables the detection of point mutations, amplifications/deletions, chromosomal abnormalities, and tumor mutational burden (TMB). (Created with BioRender.com (accessed on 20 September 2025)).

For liquid biopsy analysis, AH and peripheral blood samples were collected at the time of enucleation for cfDNA isolation (Figure 1).

cfDNA was extracted from plasma (4 mL) and AH (0.4 mL) using the QIAamp MinElute ccfDNA Kit (Qiagen, Hilden, Germany), following the manufacturer’s instructions. cfDNA quality and concentration were assessed using the Cell-free DNA ScreenTape Analysis (Agilent Technologies, Palo Alto, CA, USA) on the 4150 TapeStation System and quantified with the Qubit™ dsDNA HS Assay Kit on a Qubit 3.0 fluorometer (Invitrogen, Carlsbad, CA, USA). Library preparation was performed manually using the TruSight Oncology 500 ctDNA assay (Illumina Inc.). This hybrid-capture panel targets 523 cancer-associated genes, enabling the detection of single-nucleotide variants (SNVs), small insertions/deletions (indels), and copy number variations (CNVs) across 59 genes, as well as gene fusions within 23 genes relevant to ctDNA analysis (TSO500 ctDNA Panel information is available in Supplementary Document S1). The assay also provides quantitative assessments of tumor mutational burden (TMB) and microsatellite instability (MSI), key biomarkers of tumor immunogenicity and predictors of response to immune checkpoint inhibitors. Primary sequencing data were processed using the Illumina DRAGEN Bioinformatics Pipeline for read alignment, variant calling, and quality control. Tertiary analysis included variant annotation and prioritization using multiple public databases and in silico prediction tools, including ClinVar, OMIM, VarSome, CADD, Franklin by Genoox, LOVD, and OncoKB. Variant pathogenicity was interpreted according to American College of Medical Genetics and Genomics/Association for Molecular Pathology (ACMG/AMP) guidelines.

### 2.6. Long-Read–Whole-Genome Sequencing (LR-WGS)

To further characterize the duplication suggested by liquid biopsy, third-generation LR sequencing (TGS) was performed on genomic DNA from the patient’s peripheral blood. Sequencing was carried out using the Oxford Nanopore Technologies PromethION platform with the Ligation Sequencing Kit SQK-LSK11 (Oxford Nanopore Technologies, Oxford, UK) leveraging the LR capability of the system for high-resolution structural variant detection and assessment of potential constitutional rearrangements.

Genomic DNA yield and purity were quantified using a Qubit 4 Fluorometer (Thermo Fisher Scientific, Waltham, MA, USA) and a NanoDrop spectrophotometer. Fragment length and integrity were assessed with the Agilent Genomic DNA ScreenTape Assay on the 4150 TapeStation System (Agilent Technologies, Santa Clara, CA, USA). For library preparation, 2 µg of high-quality genomic DNA was used as input. DNA was mechanically fragmented with a Covaris g-TUBE (Covaris, Woburn, MA, USA), followed by end-repair, A-tailing, and adapter ligation using the ONT Ligation Sequencing Kit SQK-LSK114. Approximately 50 fmol of the prepared library was loaded onto a PromethION R10.4.1 flow cell. Sequencing was conducted using MinKNOW software version 24.06.15 (ONT) for a total runtime of 80 h. Live basecalling was enabled in high-accuracy (HAC) mode (basecall_model=DNA_r10.4.1_e8.2_400bps_hac@v4.3.0) with methylation-aware models for the detection of 5-mC and 5-hmC modifications. The sequencing run generated a total yield of approximately 151.8 Gb of basecalled data. Reads were aligned to the human reference genome GRCh38 (hg38) using Minimap2 version 2.24-r1122. Alignment files were processed with SAMtools version 1.20 to generate, sort, and index BAM files. Variant discovery and methylation analyses were performed using the ONT EPI2ME platform (workflow wf-human-variation), which integrates several tools optimized for long-read sequencing:

- Clair3 for single-nucleotide variants (SNVs) and small indel calling,
- Sniffles2 for structural variant (SV) detection,
- modkit for the extraction and quantification of methylation (5-mC and 5-hmC) from modBAM files,
- Straglr for the identification of short tandem repeat (STR) expansions,
- Spectre for genome-wide copy number variation (CNV) profiling.

The resulting data were used for variant annotation and integrated with cfDNA findings from the AH and plasma to achieve comprehensive genomic interpretation and correlation with clinical phenotype.

## 3. Case Description

**Figure 2:**
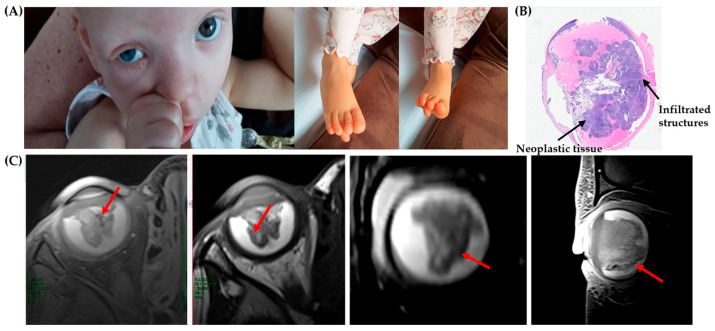
Case report. (**A**) Three-year-old female patient presenting with RB in the right eye, facial angioma, and congenital foot anomalies. (**B**) Low-power view of hematoxylin–eosin (HE)-stained retinal tissue showing invasion of RB into adjacent structures. (**C**) MRI of the right orbit, with axial images on the left side and coronal and sagittal images (on the right side), showing a placoid thickening of the retina, with no calcification and scarce enhancement, associated with subretinal exudate (red arrows).

Here, we present a 3-year-old female patient diagnosed with unilateral RB in the right eye, with no detectable germline alterations in the *RB1* gene (Figure 2). 

The patient exhibited limited verbal communication but was able to understand simple commands. Physical examination revealed a facial angioma in the glabellar region extending to the nasal bridge and philtrum, as well as mild hypertelorism (Figure 2). Congenital foot anomalies were noted: on the right foot, the third toe was positioned beneath the second and fourth toes; on the left foot, the second toe lay beneath the third.

The patient was conceived through assisted reproductive technology (ART), the couple’s only pregnancy. She was born at 37 + 5 weeks of gestation (late preterm) via cesarean section due to placenta previa, with a birth weight of 2630 g. Maternal age at conception was 52 years. Family history was largely unremarkable, except for a case of RB in a child of the father’s second cousin. She was referred to our center for molecular analysis to identify the genetic cause of the disease. The patient underwent a comprehensive radiological assessment, including ultrasound of the pilonidal sinus and magnetic resonance imaging (MRI) of the brain and orbits (Figure 2). The investigation revealed a widely infiltrative intraocular mass without evidence of extraocular or intracranial extension (Figure 2). Based on imaging and clinical features, conservative management was not feasible, and enucleation was indicated.

Histopathological examination of the enucleated globe (Figure 2) showed poorly differentiated neuroblastic cells with hyperchromatic nuclei and scant cytoplasm, occasionally forming Homer Wright rosettes, with areas of necrosis.

Following enucleation, the patient received six cycles of high-risk chemotherapy with vincristine and cyclophosphamide according to institutional protocols for advanced RB. This was followed by external-beam radiotherapy to the right orbit and the optic nerve stump. Post-radiation effects included mild periorbital dermatitis, managed with topical therapy, and intermittent orbital edema with mucous discharge; repeated microbiologic cultures were negative for infection. Given the high-risk histopathologic features (pT4 with optic nerve involvement), she subsequently underwent autologous stem-cell transplantation as consolidation therapy after high-dose chemotherapy. During pre-transplant evaluation, hepatitis B virus (HBV) seropositivity was detected, and she received antiviral prophylaxis throughout chemotherapy and for 6–12 months thereafter. The associated anomalies—facial hemangioma and toe malformations—did not require intervention and remain under clinical surveillance. The patient continues regular oncologic follow-up every six months and remains in complete remission, with no evidence of local recurrence or systemic metastasis.

### 3.1. Results

#### 3.1.1. Clinical Exome Sequencing (CES) and MS-MLPA Results

Conventional germline analyses for RB etiologies—including CES and MS-MLPA for copy number variations and MS-PCR of the *RB1* promoter—revealed no evidence of *RB1* alterations (MS-MLPA profiles are shown in Supplementary Figure S1). These results excluded the presence of classical germline *RB1* defects typically responsible for hereditary RB, supporting a non-heritable (sporadic) origin.

#### 3.1.2. Liquid Biopsy Results

**Figure 3:**
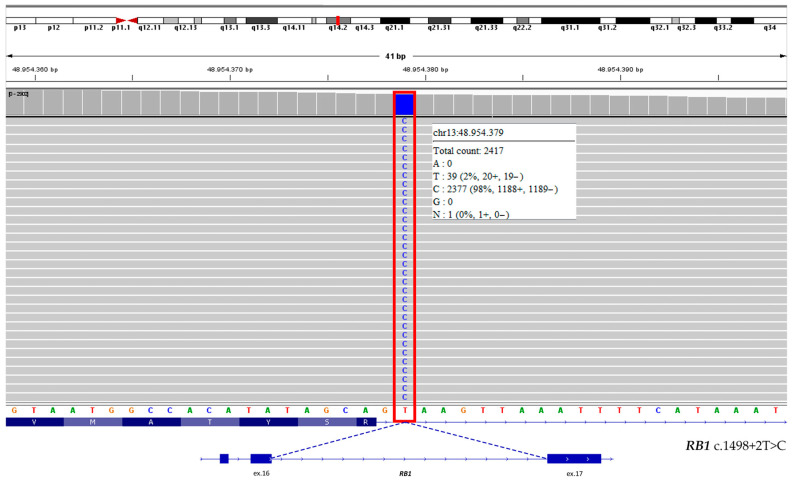
Somatic *RB1* splice-site variant identified in aqueous humor cfDNA. Integrative Genomics Viewer (IGV) visualization showing the somatic c.1498+2T>C substitution (in the red box) at the exon 16–intron 16 boundary of *RB1*. This single-nucleotide change disrupts the invariant GT dinucleotide at the 5′ splice donor site, which is essential for accurate pre-mRNA splicing. The affected exon encodes part of the A domain within the pRB pocket region, critical for E2F transcription factor binding and regulation of cell cycle progression. Read alignments and splice junctions were visualized using the Integrative Genomics Viewer (IGV, version 2.19.6; Robinson et al., 2011 [17]; Thorvaldsdóttir et al., 2013 [18]).

Accordingly, liquid biopsy analysis comparing cfDNA from AH and plasma revealed a somatic *RB1* splice-site variant c.1498+2T>C with a VAF of 98.5% (Figure 3) only in AH, suggesting tumor biallelic inactivation.

This pattern may result either from a deletion of the wild-type allele or from a deletion and subsequent duplication of the mutant allele. Both scenarios could explain the apparent homozygosity of the variant and are consistent with the two-hit model of *RB1* inactivation. Liquid biopsy analysis also detected notable CNVs: gains in *FGF14* (chr13:102,375,179–103,054,030) and *LAMP1* (chr13:113,951,748–113,977,608) were observed in AH, with fold changes greater than 1.5, and confirmed in plasma (Table 1). These findings indicated the presence of a germinal duplication in the distal region of chromosome 13q, where *FGF14* and *LAMP1* are located (13q33.1 and 13q34 cytobands) (Table 1).

AH cfDNA analysis further revealed multiple SCNAs restricted to the tumor, including duplications in *MDM4*, *ALK*, *CCND3*, and *BRAF*, as well as deletions in *TFRC*, *FGF3*, *FGF9*, and *BRCA2* (Table 1). Concerning SNVs, combined AH/plasma cfDNA-NGS analysis confirmed that RB tumors harbor stable genomes with a limited number of mutations (Table 2).

Two variants, one in *FGFR4* and the other in *FANCD2*, showed comparable VAFs in AH and plasma, suggesting they likely represent germline polymorphisms. The other three variants—located in *PMS2*, *STK11*, and *CHEK2*—were classified as tumor-associated VUSs (Table 2). The uncertain *BRCA2* variant (c.7871A>G, p.Tyr2624Cys) was detected in both AH and plasma, with significant differences in VAF between the two fluids (1.3% in AH vs. 45% in plasma) (Table 2).

#### 3.1.3. Long-Read–Whole-Genome Sequencing (LR-WGS) Results

**Figure 4:**
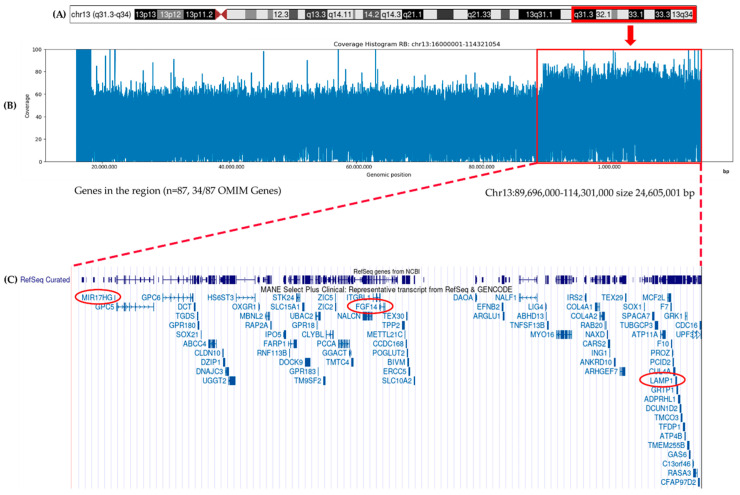
Structural characterization of the 13q duplication in the RB case. (**A**) Schematic representation of chromosome 13, with the duplicated region identified in the patient highlighted in red. (**B**) Coverage histogram from raw LR-WGS data showing increased read depth across the duplicated segment. (**C**) The amplified region was mapped using the UCSC Human Genome Browser (hg38; December 2013 release, http://genome.ucsc.edu (accessed on 20 September 2025)). Genes located within this interval are indicated (*n* = 87; listed in Supplementary Document S2). *FGF14* and *LAMP1* genes (also found duplicated in both AH and plasma liquid biopsy) are shown in red, together with *MIR17HG*, an oncogenic lncRNA previously implicated in RB.

Oxford Nanopore TGS LR-WGS performed on genomic DNA confirmed the presence of a large germline duplication (Figure 4) encompassing the distal long arm of chromosome 13 (13q31.3–q34).

**Figure 5:**
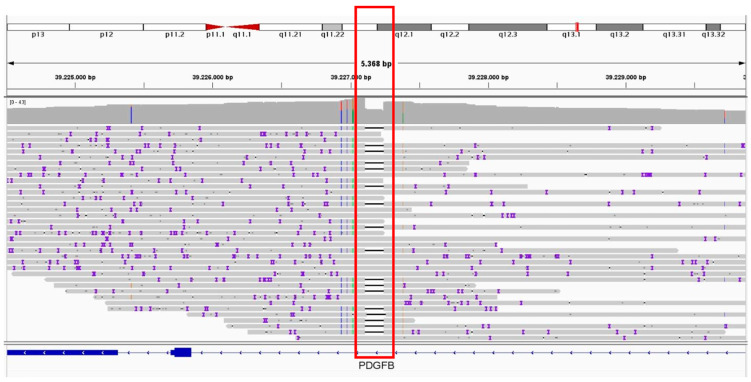
IGV visualization of a germline intronic deletion in the *PDGFB* gene (chr22:39,227,243–39,227,110 GRCh38) identified through ONT LR-WGS. The upper coverage track displays the read-depth profile together with small colored bars (A = green, C = blue, G = yellow, T = red), which represent aggregated mismatches occurring at a given genomic position across all aligned reads. In the alignment track below, the purple marks indicate individual base-level mismatches within each long read, mainly reflecting the intrinsic sequencing error rate of Oxford Nanopore reads. The black bar within the read alignments corresponds to a 134-bp deleted genomic segment in intron 5, highlighted in the red box. LR-WGS enables direct visualization of this structural variant, which would be more challenging to detect using short-read WGS approaches. Read alignments were displayed using the Integrative Genomics Viewer (IGV, version 2.19.6; Robinson et al., 2011 [17]; Thorvaldsdóttir et al., 2013 [18]).

This rearrangement is consistent with distal 13q duplication syndrome and accounts for the patient’s phenotypic features, including facial hemangioma and digital foot anomalies. WGS analysis mapped the 24.6 Mb duplication on chromosome 13q (chr13:89,696,000–114,301,000), including 87 genes (listed in Supplementary Document S2). The duplicated region includes the oncogenic lncRNA *MIR17HG*, which has been implicated in RB pathogenesis. In addition, LR-WGS performed on germline DNA confirmed the heterozygous missense *BRCA2* variant (p.Tyr2624Cys, exon 17) and revealed a 134-bp deletion in intron 5 of the *PDGFB* gene (chr22:39,227,243 –39,227,110) (Figure 5).

## 4. Discussion

A key element of this case is that aqueous humor (AH) cfDNA analysis proved essential for establishing the molecular diagnosis. In RB, direct tumor biopsy is precluted, and genomic characterization frequently relies on AH-derived cfDNA as a minimally invasive surrogate for tumor tissue. In our patient, AH testing was the only approach capable of revealing the somatic *RB1* second hit, thereby defining complete biallelic inactivation in the absence of germline alterations. These findings highlight how AH cfDNA can provide clinically actionable information at the time of diagnosis—well before enucleation—and underscore its central role within the modern molecular workup of RB.

### 4.1. Molecular Findings and RB1 Pathogenic Variant

Liquid biopsy in cfDNA derived from AH identified the *RB1* somatic variant c.1498+2T>C, absent in both plasma cfDNA and germline DNA. This variant has a VAF of 98.5% in AH, in accordance with the two-hit model of tumorigenesis and affects the canonical donor splice site immediately downstream of exon 16. Differentiating between germline and somatic *RB1* mutations is clinically crucial, since patients carrying germline alterations require lifelong surveillance for both secondary ocular tumors and late-onset systemic malignancies. Conversely, when molecular testing excludes a constitutional mutation, the risk of these additional neoplasms is markedly reduced [19].

### 4.2. Somatic Copy Number Alterations (SCNAs) and Oncogenic Drivers

AH liquid biopsy also showed several tumor gene SCNAs, suggesting the presence of larger genomic rearrangements that have been recurrently found in RB (1q, 2p, 6p gain and 13q loss) (Table 1) [3]. Copy-number gain of *MDM4* has been identified in approximately 65% of retinoblastoma cases, correlating with elevated transcript and protein levels [20,21]. Further analyses have shown that MDM4 overexpression can occur independently of gene amplification, suggesting additional regulatory mechanisms controlling its transcriptional and post-transcriptional activity [22]. This transcriptional profile aligns with the elevated MDM4 protein expression observed in RB cell lines and in primary human orthotopic xenograft models [21]. *ALK* amplification occurs more frequently in non-responsive RBs and is significantly associated with optic nerve invasion [23]. Importantly, *ALK* encodes a therapeutically targetable kinase, and the reported genomic gains offer early evidence supporting the potential of kinase-directed therapies in RB [23]. The presence of *CCND3* duplication in AH cfDNA may serve as an indicator of chromosome 6p gain, a hallmark of aggressive disease and poor ocular survival in RB [24]. However, the other TSO500 genes located on chromosome 6p showed no CNVs, excluding this correlation. Although SCNAs detection through AH cfDNA is now well established, such alterations are rarely observed in plasma cfDNA (Table 2) because of the low fraction of tumor-derived DNA. This difference was clearly demonstrated by Berry et al. (2017), who directly compared matched AH and plasma samples [13]_._

### 4.3. Additional Molecular Findings

The analysis also revealed a TMB for the humor sample of 4.1. Low TMB are generally considered less likely to respond to immunotherapy because they produce fewer neoantigens. This explains why immunotherapy is less commonly used in RB treatment compared to other cancers. Liquid biopsy also detected a VUS in *BRCA2* (c.7871A>G p.Tyr2624Cys) with marked discrepancy in the VAF between plasma cfDNA (45%) and AH cfDNA (1.3%). This variant was confirmed in the heterozygous state by WGS, establishing its germline origin. Copy number analysis revealed a somatic deletion in *BRCA2* spanning exons 3–25 (chr13:32,890,596–32,972,909; 82,314 bp), encompassing the region harboring the *BRCA2* point mutation on exon 17 (p.Tyr2624Cys). These data support the hypothesis that the deletion occurred in *cis* with the germline variant, leading to the selective loss of the mutant allele in the tumor clone.

### 4.4. Chromosomal Duplication and Rare Genetic Events

The 13q duplication suggested by liquid biopsy and characterized by WGS aligns with a rare chromosomal anomaly syndrome, characterized by intellectual disability, psychomotor delay, craniofacial dysmorphisms, and hemangiomas [24,25] (Orphanet reference, ORPHA:96105), many of which are observed in our patient. Interestingly, the rearrangement includes at one extremity the lncRNA miR-17-92a-1 cluster host gene (*MIR17HG*) that exerts oncogenic effects in RB via the miR-155-5p/HIF-1α axis [26] and the hsa-mir-425-5p/MDM2 pathway [27]. This cluster has been implicated in promoting tumor cell proliferation and invasion [28], underscoring its biological relevance in RB progression. LR-WGS analysis also revealed a 134-bp deletion in intron 5 of the *PDGFB* gene (chr22:39,227,243–39,227,110). This *PDGFB* deletion is currently regarded as a rare intronic uncertain variant. A similar alteration has been reported in a familial case and in one sporadic case of meningioma [29,30]. Although its functional impact remains uncertain, the presence of this alteration may guide meningioma-specific surveillance.

### 4.5. Limitations of the Study

This study has some limitations that should be acknowledged. Although a tumor specimen became available after enucleation, direct tumor DNA analysis could not be performed because the sample was formalin-fixed and paraffin-embedded, precluding high-quality genomic extraction. Therefore, a direct comparison between tumor tissue and AH cfDNA was not feasible. Nonetheless, AH cfDNA provides a broader and more integrated representation of the tumor genome, whereas tissue biopsies may reflect only clone-limited alterations. This concept is supported by several studies demonstrating that AH cfDNA accurately mirrors the somatic genomic landscape of RB and serves as a validated minimally invasive molecular tool for diagnosis, prognostic assessment, and therapeutic monitoring [31,32].

## 5. Conclusions

In conclusion, we describe a previously unreported case of distal 13q trisomy coexisting with RB, associated with hemangioma, mild hypertelorism, and digital foot anomalies. To our knowledge, this represents the first documented instance of a distal 13q duplication identified in an RB patient and the first case delineated by LR sequencing (ONT), enabling precise breakpoint mapping and detailed characterization of the underlying genomic architecture. These results contribute to refining the phenotypic and molecular spectrum of the distal 13q33–q34 region and strengthen genotype–phenotype correlations in this rare chromosomal disorder. Our findings underscore the diagnostic value of integrating high-resolution genomic approaches. The combined use of NGS-based liquid biopsy on cfDNA from AH and plasma, together with LR-WGS, proved effective in simultaneously detecting somatic *RB1* inactivation (“two-hit” pattern), germline susceptibility linked to the 13q duplication, and tumor clonal evolution involving CNVs and SNVs. Moreover, LR-WGS identified an additional 134-bp intronic deletion in *PDGFB*, further illustrating how LR sequencing can uncover subtle structural variants potentially relevant to disease susceptibility. This comprehensive strategy expands current diagnostic paradigms and highlights the potential of multi-omic liquid biopsy and LR sequencing to resolve diagnostically elusive and genetically heterogeneous cases.

## Figures and Tables

**Table 1:** Patient CNVs in Aqueous Humor (AH) and Plasma cfDNA Liquid Biopsy Analysis.

HUMOR-cfDNA NGS Liquid Biopsy					PLASMA-cfDNA NGS Liquid Biopsy				
CNV					CNV				
Gene	Chr	Cytoband	Interval	Fold Change	Gene	Chr	Cytoband	Interval	Fold Change
*MDM4*	1	1q32.1	204,485,505–204,526,342	1.4	*MDM4*	1	1q32.1	/	/
*ALK*	2	2p23.2	29,416,088–30,143,527	1.3	*ALK*	2	2p23.2	/	/
*TFRC*	3	3q29	195,776,752–195,806,640	0.5	*TFRC*	3	3q29	/	/
*CCND3*	6	6p21.2	41,903,676–42,014,927	1.4	*CCND3*	6	6p21.2	/	/
*BRAF*	7	7q34	140,434,395–140,624,505	1.1	*BRAF*	7	7q34	/	/
*FGF3*	11	11q13.3	69,623,035–69,635,441	0.8	*FGF3*	11	11q13.3	/	/
*FGF9*	13	13q12.11	22,245,512–22,278,213	0.6	*FGF9*	13	13q12.11	/	/
*BRCA2*	13	13q13.1	32,890,596–32,972,909	0.8	*BRCA2*	13	13q13.1	/	/
*FGF14*	13	13q33.1	102,375,179–103,054,030	1.7	*FGF14*	13	13q33.1	/	1.3
*LAMP1*	13	13q34	113,951,748–113,977,608	1.8	*LAMP1*	13	13q34	/	1.3

**Table 2:** Patient SNVs in Aqueous Humor (AH) and Plasma cfDNA Liquid Biopsy Analysis.

HUMOR-cfDNA NGS Liquid Biopsy							PLASMA-cfDNA NGS Liquid Biopsy						
SNV							SNV						
Gene	Chr	Variant	CADD	VAF %	Read Depth	ACMG Class	Gene	Chr	Variant	CADD	VAF %	Read Depth	ACMG Class
*FANCD2*	3	c.573C>G p.(Ser191Arg)	9.7	49	1181	VUS	*FANCD2*	3	c.573C>G p.(Ser191Arg)	9.7	49	604	VUS
*FGFR4*	5	c.1162G>A p.(Gly388Arg)	23.6	48	1942	Benign	*FGFR4*	5	c.1162G>A p.(Gly388Arg)	23.6	48	602	Benign
*PMS2*	7	c.20C>G p.(Ser7Trp)	13.38	0.8	891	VUS	*PMS2*	7	/	/	/	/	/
*BRCA2*	13	c.7871A>G p.(Tyr2624Cys)	26.7	1.3	1051	VUS	*BRCA2*	13	c.7871A>G p.(Tyr2624Cys)	26.7	45	596	VUS
*RB1*	13	c.1498+2T>C	32	98.5	1177	Pathogenic	*RB1*	13	/	/	/	/	/
*STK11*	19	c.1069G>A p.(Glu357Lys)	24	3.9	1294	VUS	*STK11*	19	/	/	/	/	/
*CHEK2*	22	c.1117A>G p.(Lys373Glu)	27.7	3.5	1888	VUS	*CHEK2*	22	/	/	/	/	/

Abbreviation: ACMG Class, American College of Medical Genetics and Genomics Classification; AH, Aqueous Humor; CADD, Combined Annotation Dependent Depletion; VAF, Variant Allele Frequency; VUS, Variant of Uncertain Significance.

## Data Availability

The original contributions of this study are included in the article and its Supplementary Material. Further inquiries can be directed to the corresponding author.

## Supplementary Materials

The following supporting information can be downloaded at: https://www.mdpi.com/article/10.3390/genes16121399/s1, Figure S1 MLPA analysis of genomic DNA from the patient with retinoblastoma. The assay did not reveal duplications or deletions within the RB1 gene. Methylation-specific probes for the promoter regions CpG106 and CpG85 also showed normal profiles, excluding epigenetic alterations at these sites. Document S1: Illumina TruSight Oncology 500ctDNA—Panel of genes. Document S2: 13q33.1-13q34 duplication analysis—list of genes mapped.

## Abbreviations

The following abbreviations are used in this manuscript:

AH	Aqueous humor
ACMG	American College of Medical Genetics and Genomics
AMP	Association for Molecular Pathology
ART	Assisted reproductive technology
AURKA	Aurora kinase A
cfDNA	Cell-free DNA
ctDNA	Circulating tumor DNA
CNV	Copy number variation
HAC	High accuracy
HBV	Hepatitis B Virus
HE	Hematoxylin/Eosin
IGV	Integrative Genomics Viewer
INDEL	Insertion/deletion
lnRNA	Long non-coding RNA
LR	Long read
MRI	Magnetic resonance image
MSI	Microsatellite instability
MS-MLPA	Methylation-Specific Multiplex ligation probe amplification
NGS	Next-generation sequencing
ONT	Oxford Nanopore Technology
pRB	Retinoblastoma Protein
RB	Retinoblastoma
SCNA	Somatic copy number alteration
SNV	Single-nucleotide variant
STR	Short tandem repeat
SV	Structural variant
TGS	Third-generation sequencing
TMB	Tumor mutational burden
TSO	TruSight Oncology
VAF	Variant allele frequency
VUS	Variant of uncertain significance
WES	Whole-exome sequencing
WGS	Whole-genome sequencing

## References

[1] Chen J., Cao X., Xu S., Chen X., Xie R., Ye G., Zhang Y., Huang S., Shen X., Xiao Y. (2024). Global, Regional, and National Burden of Retinoblastoma in Infants and Young Children: Findings from the Global Burden of Disease Study 1990–2021. EClinicalMedicine.

[2] Dimaras H., Corson T.W., Cobrinik D., White A., Zhao J., Munier F.L., Abramson D.H., Shields C.L., Chantada G.L., Njuguna F. (2015). Retinoblastoma. Nat. Rev. Dis. Primers.

[3] Dimaras H., Khetan V., Halliday W., Orlic M., Prigoda N.L., Piovesan B., Marrano P., Corson T.W., Eagle R.C., Squire J.A. (2008). Loss of RB1 induces non-proliferative retinoma: Increasing genomic instability correlates with progression to retinoblastoma. Hum. Mol. Genet..

[4] Guzmán F., Fazeli Y., Khuu M., Salcido K., Singh S., Benavente C.A. (2020). Retinoblastoma Tumor Suppressor Protein Roles in Epigenetic Regulation. Cancers.

[5] Iacovacci J., Brough R., Moughari F.A., Alexander J., Kemp H., Tutt A.N.J., Natrajan R., Lord C.J., Haider S. (2025). Proteogenomic discovery of RB1-defective phenocopy in cancer predicts disease outcome, response to treatment, and therapeutic targets. Sci. Adv..

[6] Flores M., Goodrich D.W. (2022). Retinoblastoma Protein Paralogs and Tumor Suppression. Front. Genet..

[7] Lyu J., Yang E.J., Zhang B., Wu C., Pardeshi L., Shi C., Mou P.K., Liu Y., Tan K., Shim J.S. (2020). Synthetic lethality of RB1 and aurora A is driven by stathmin-mediated disruption of microtubule dynamics. Nat. Commun..

[8] Liao Q., Yang J., Shi H., Mengjiang R., Li Y., Zhang Q., Wen X., Ge S., Chai P., Fan X. (2025). Aurora A Kinase Inhibition Is Synthetic Lethal With the Activation of MYCN in Retinoblastoma. Investig. Ophthalmol. Vis. Sci..

[9] O’Leary B., Finn R.S., Turner N.C. (2016). Treating cancer with selective CDK4/6 inhibitors. Nat. Rev. Clin. Oncol..

[10] Corson T.W., Gallie B.L. (2007). One hit, two hits, three hits, more? Genomic changes in the development of retinoblastoma. Genes Chromosomes Cancer.

[11] Rushlow D.E., Mol B.M., Kennett J.Y., Yee S., Pajovic S., Thériault B.L., Prigoda-Lee N.L., Spencer C., Dimaras H., Corson T.W. (2013). Characterisation of retinoblastomas without RB1 mutations: Genomic, gene expression, and clinical studies. Lancet Oncol..

[12] McEvoy J., Nagahawatte P., Finkelstein D., Richards-Yutz J., Valentine M., Ma J., Mullighan C., Song G., Chen X., Wilson M. (2014). RB1 gene inactivation by chromothripsis in human retinoblastoma. Oncotarget.

[13] Berry J.L., Xu L., Murphree A.L., Krishnan S., Stachelek K., Zolfaghari E., McGovern K., Lee T.C., Carlsson A., Kuhn P. (2017). Potential of aqueous humor as a surrogate tumor biopsy for retinoblastoma. JAMA Ophthalmol..

[14] Berry J.L., Xu L., Polski A., Jubran R., Kuhn P., Kim J.W., Hicks J. (2020). Aqueous humor is superior to blood as a liquid biopsy for retinoblastoma. Ophthalmology.

[15] Gerrish A., Jenkinson H., Cole T. (2021). The impact of cell-free DNA analysis on the management of retinoblastoma. Cancers.

[16] Xu L., Polski A., Prabakar R.K., Reid M.W., Chevez-Barrios P., Jubran R., Kim J.W., Kuhn P., Cobrinik D., Hicks J. (2020). Chromosome 6p amplification in aqueous humor cell-free DNA is a prognostic biomarker for retinoblastoma ocular survival. Mol. Cancer Res..

[17] Robinson J.T., Thorvaldsdóttir H., Winckler W., Guttman M., Lander E.S., Getz G., Mesirov J.P. (2011). Integrative Genomics Viewer. Nat. Biotechnol..

[18] Thorvaldsdóttir H., Robinson J.T., Mesirov J.P. (2013). Integrative Genomics Viewer (IGV): High-performance genomics data visualization and exploration. Brief. Bioinform..

[19] Gerrish A., Stone E., Clokie S., Ainsworth J.R., Jenkinson H., McCalla M., Hitchcott C., Colmenero I., Allen S., Parulekar M. (2019). Non-invasive diagnosis of retinoblastoma using cell-free DNA from aqueous humour. Br. J. Ophthalmol..

[20] Laurie N.A., Donovan S.L., Shih C.S., Zhang J., Mills N., Fuller C., Teunisse A., Lam S., Ramos Y., Mohan A. (2006). Inactivation of the p53 pathway in retinoblastoma. Nature.

[21] McEvoy J., Flores-Otero J., Zhang J., Nemeth K., Brennan R., Bradley C., Krafcik F., Rodriguez-Galindo C., Wilson M., Xiong S. (2011). Coexpression of normally incompatible developmental pathways in retinoblastoma genesis. Cancer Cell.

[22] McEvoy J., Ulyanov A., Brennan R., Wu G., Pounds S., Zhang J., Dyer M.A. (2012). Analysis of MDM2 and MDM4 single nucleotide polymorphisms, mRNA splicing and protein expression in retinoblastoma. PLoS ONE.

[23] Jeyaprakash K., Kumaran M., Kim U., Santhi R., Muthukkaruppan V., Devarajan B., Vanniarajan A. (2024). Investigating druggable kinases for targeted therapy in retinoblastoma. J. Hum. Genet..

[24] Ioan D.M., Vermeesch J., Fryns J.P. (2005). Terminal distal 13q trisomy due to de novo dup(13)(q32→qter). Genet. Couns..

[25] Rogers J.F. (1984). Clinical delineation of proximal and distal partial 13q trisomy. Clin. Genet..

[26] Yan J., Deng Y.X., Cai Y.L., Cong W.D. (2022). LncRNA MIR17HG promotes the proliferation, migration, and invasion of retinoblastoma cells by up-regulating HIF-1α expression via sponging miR-155-5p. Kaohsiung J. Med. Sci..

[27] Wang Z., Liang X., Yi G., Wu T., Sun Y., Zhang Z., Fu M. (2024). Bioinformatics analysis proposes a possible role for long noncoding RNA MIR17HG in retinoblastoma. Cancer Rep..

[28] Kandalam M.M., Beta M., Maheswari U.K., Swaminathan S., Krishnakumar S. (2012). Oncogenic microRNA 17-92 cluster is regulated by epithelial cell adhesion molecule and could be a potential therapeutic target in retinoblastoma. Mol. Vis..

[29] Bolger G.B., Stamberg J., Kirsch I.R., Hollis G.F., Schwarz D.F., Thomas G.H. (1985). Chromosomal Translocation t(14;22) and Oncogene (c-sis) Variant in a Pedigree with Familial Meningioma. N. Engl. J. Med..

[30] Smidt M., Kirsch I., Ratner L. (1990). Deletion of Alu Sequences in the Fifth c-sis Intron in Individuals with Meningiomas. J. Clin. Invest..

[31] Xu L., Kim M.E., Polski A., Prabakar R.K., Shen L., Peng C.C., Reid M.W., Chévez-Barrios P., Kim J.W., Shah R. (2021). Establishing the Clinical Utility of ctDNA Analysis for Diagnosis, Prognosis, and Treatment Monitoring of Retinoblastoma: The Aqueous Humor Liquid Biopsy. Cancers.

[32] Mendes T.B., Oliveira I.D., Gamba F.T., Lima F.T., Morales B.F.S.C., Macedo C.R.D., Teixeira L.F., de Toledo S.R.C. (2025). Retinoblastoma: Molecular Evaluation of Tumor Samples, Aqueous Humor, and Peripheral Blood Using a Next-Generation Sequence Panel. Int. J. Mol. Sci..

