# Supplementary material for: A Complex Case of Retinoblastoma Solved by the Combined Approach of Humor/Plasma cfDNA-NGS and LR-WGS
Source: Genes (Basel). 2025 Nov 22;16(12):1399. doi: 10.3390/genes16121399 (PMC12732515; doi:10.3390/genes16121399)
Supplement: Supplementary file 1 [file genes-16-01399-s001.zip › Document S2_Genes.pdf]

13q33.1-13q34 duplication analysis – List of genes mapped

The duplication spans approximately 24 Mb on chromosome 13q31.3–q34 and includes about 87 genes, of which 34 are annotated in OMIM. The genes in this interval are: *MIR17HG, CPC6, HS6ST3, STK24, ZIC5, ITGBL1, DAOA, NALF1, IRS2, TEX29, MCF2L, GPC5, DCT, OXGR1, SLC15A1, ZIC2, FGF14, EFNB2, LIG4, COL4A1, SOX1, F7, TGDS, MBNL2, UBAC2, NALCN, TEX30, ARGLU1, ABHD13, COL4A2, SPACA7, GRK1, GPR180, RAP2A, GPR18, TPP2, TNFS13B, RAB20, TUBGCP3, CDC16, SOX21, IPO5, CLYBL, METTL21C, MYO16, NAXD, ATP11A, UPF3A, ABCC4, FARP1, PCCA, CCDC168, CARS2, F10, CLDN10, RNF113B, GGACT, POGLUT2, ING1, PROZ, DZIP1, DOCK9, TMTC4, BIVM, ANKRD10, PCID2, DNAJC3, GPR183, ERCC5, ARGEF7, CUL4A, UGGT2, TM9SF2, SLC10A2, LAMP1, GRTP1, ADPRHL1, DCUN1D2, TMCO3, TFDP1, ATP4B, TMEM255B, GAS6, C13orf46, RASA3, CFAP97D2*. Human Genome Browser UCSC, http://genome.cse.ucsc.edu, release December 2013.
