# Supplementary figures and images for: A Complex Case of Retinoblastoma Solved by the Combined Approach of Humor/Plasma cfDNA-NGS and LR-WGS
Source: Genes (Basel). 2025 Nov 22;16(12):1399. doi: 10.3390/genes16121399 (PMC12732515; doi:10.3390/genes16121399)

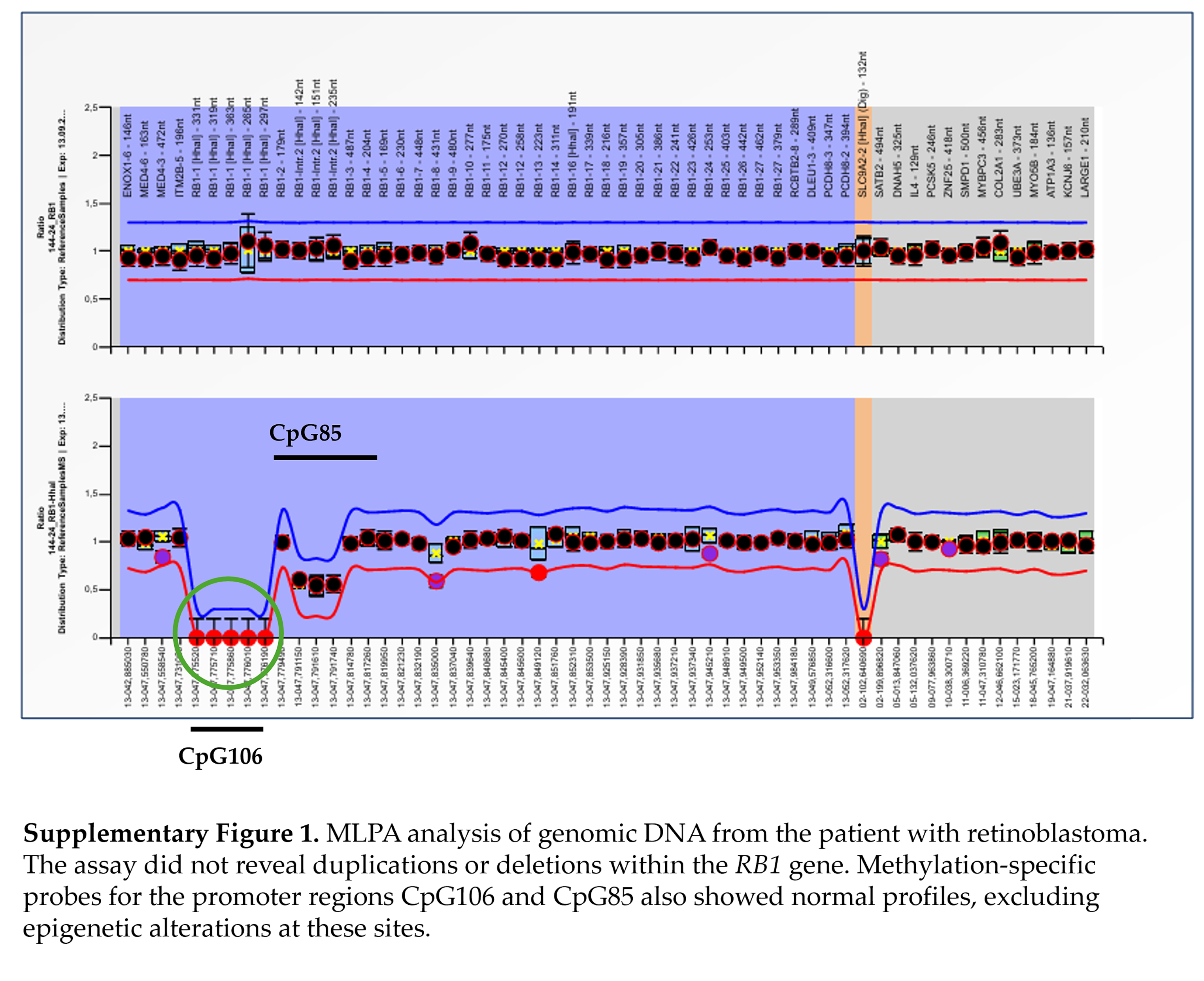

Supplement: Supplementary file 1 [file genes-16-01399-s001.zip › Figure S1_Genes.png]
